# Cellular Composition and Contribution of Tertiary Lymphoid Structures to Tumor Immune Infiltration and Modulation by Radiation Therapy

**DOI:** 10.3389/fonc.2018.00256

**Published:** 2018-07-09

**Authors:** Gaël Boivin, Pradeep Kalambaden, Julien Faget, Sylvie Rusakiewicz, Pierre Montay-Gruel, Etienne Meylan, Jean Bourhis, Guy Lesec, Marie-Catherine Vozenin

**Affiliations:** ^1^Radio-Oncology Laboratory, Department of Oncology, Lausanne University Hospital, University of Lausanne, Lausanne, Switzerland; ^2^School of Life Sciences, Swiss Institute for Experimental Cancer Research, Ecole Polytechnique Fédérale de Lausanne, Lausanne, Switzerland; ^3^Center of Experimental Therapies (CTE), Department of Oncology, Lausanne University Hospital, University of Lausanne, Lausanne, Switzerland; ^4^Radio-Oncology Service, Department of Oncology, Lausanne University Hospital, University of Lausanne, Lausanne, Switzerland; ^5^Unilabs, Epalinges, Switzerland

**Keywords:** medullary breast carcinoma, radiation therapy, tertiary lymphoid structures, microenvironment, KP model

## Abstract

Immune-based anti-cancer strategies combined with radiation therapy (RT) are actively being investigated but many questions remain, such as the ideal treatment scheme and whether a potent immune response can be generated both locally and systemically. In this context, tumor-associated tertiary lymphoid structures (TLS) have become a subject of research. While TLS are present in several types of cancer with strong similarities, they are especially relevant in medullary breast carcinoma (MBC). This suggests that MBC patients are ideally suited for investigating this question and may benefit from adapted therapeutic options. As RT is a corner-stone of MBC treatment, investigating interactions between RT and TLS composition is also clinically relevant. We thus first characterized the lymphoid structures associated with MBC in a patient case report and demonstrated that they closely resemble the TLS observed in a genetical mouse model. In this model, we quantitatively and qualitatively investigated the cellular composition of the tumor-associated TLS. Finally, we investigated TLS regulation after hypo-fractionated RT and showed that RT induced their acute and transient depletion, followed by a restoration phase. This study is the first work to bring a comprehensive and timely characterization of tumor-associated TLS in basal conditions and after RT. It highlights cellular targets (i.e., Tregs) that could be selectively modulated in subsequent studies to optimize anti-tumor immune response. The study of TLS modulation is worth further investigation in the context of RT and personalized medicine.

## Introduction

Medullary breast carcinoma (MBC) is a unique and curious subtype of infiltrating ductal carcinoma first described in 1945 (1). According to histological risk-grading procedures, it is classified as medium to high grade and often exhibits basal-like biological features and a triple negative molecular status. In 1977, Ridolfi et al. published a study with 10 years longitudinal follow-up on MBC and showed that only 13.5% of MBC patients had LN metastasis. From this seminal study, it appeared that MBC was less prone to metastatic development and consequently had a relatively good prognosis, despite its epithelial grade and molecular profile status. It was speculated early on that this paradox was linked to the typical lymphoplasmatic intra-tumor infiltration and/or the presence of tertiary lymphoid structures (TLS) at the vicinity of MBC (2). However, recent work has challenged this notion of a favorable prognosis (3) and radio-chemotherapy remains the standard of care for these patients (4), at the costly risk, however, of over-treating young patients.

In the current increasing effort to understand and harness anti-tumor immune responses (5–9), TLS have recently become a focal point for research. The presence of TLS has indeed been correlated with a good prognosis value in various primary tumors, such as triple negative breast carcinoma, colorectal cancer, gastric cancer, non-small cell lung carcinoma, melanoma, oral squamous cell carcinoma, pancreatic carcinoma, renal cell carcinoma, and correlated with a negative prognostic value in hepatocellular cancer (10). Further work also identified biomarkers that correlated with TLS quality and prognosis value such as DC-Lamp high or CD83+ that identify mature dendritic cells; PNAd+ high endothelium venules with the presence of CD3 T cells and expression of the CXCL13 cytokine. Although these biomarkers appear to be a key for TLS formation, they are not specific to a primary tumor type and can be found across breast, lung, and colon carcinoma (11, 12). More recently, an extensive characterization of gene expression in tumors associated with TLS has been performed and enhanced our understanding about TLS formation (10). Modulation of TLS was suggested to be an attractive option to improve anti-tumor immunity *in situ* or enhance the efficacy of current immunomodulators, but these novel options have not yet been translated into the clinical setting.

In addition, understanding how TLS are modulated after anti-cancer treatment is still needed but the experimental models available to study TLS function are limited. Although the presence of TLS was reported in a pre-clinical mouse model of melanoma (13) and correlated with extended survival in another pancreatic cancer model (14), the *Kras^Lox-STOP-LOX-G12D/WT^; p53^Flox/Flox^* (KP) mouse model of lung adenocarcinoma is so far the only model where functionality of TLS was suggested (15). In KP/OVA mice, anti-tumor-specific adoptively transferred T-OVA cells were shown to proliferate in TLS. Therefore, this model is interesting in terms of characterizing TLS and investigating their response to an anti-cancer treatment such as radiation therapy (RT). RT is able to modulate tumor immune infiltrate (16–18) as: it induces immunogenic tumor cell death that favors tumor antigen release and processing; it alters the local cytokine/chemokine balance to favor antigen processing, DC maturation, and immune cell functionality; and it reorganizes the microenvironment by depleting/recruiting/polarizing adequate immune cells locally (19). However, to our knowledge, no studies have investigated the response of TLS to radiation in order to establish relevant schemes of treatment applicable to this well-defined group of patients.

The goal of this work was to provide new insights to the cellular composition of TLS and their contribution to anti-tumor immune infiltration. To do this, we first highlight two histo-pathologically related cases of basal breast cancer which differ by the presence of proliferating TLS versus nonspecific inert lymphoid aggregates. Then to further investigate TLS composition, we used KP mice which are the only autochthonous mouse model known to display functional TLS to date (15). Finally, we assessed combined contribution of TLS and tumors to immune infiltrate during the time course of RT.

## Materials and Methods

### Human Histopathological Samples

Surgical samples of two patients with breast cancer were analyzed after HE staining by histopathology. Tumors were located inside the mammary gland away from any lymph node. For diagnosis of MBC and basal carcinoma, characterization of molecular subtypes was performed using routine staining (ER, PR, HER2, Ecad, cytokeratin, and Ki-67). In addition, immune infiltrates were analyzed using CD4, CD8, CD20, PD1, and FoxP3. The number of CD8 and CD4 positive cells was quantified under microscope. The two patients were treated at “Hospital Riviera group,” VD Switzerland. They signed an informed consent and agreed to the use of their clinical data and pathology into a scientific publication.

### Human Immunohistochemistry Analysis

For the multiplexed staining, 4 µm formalin-fixed paraffin-embedded sections from breast tumor were stained by automated immunostainer (DISCOVERY ULTRA, Ventana Roche). Heat-induced antigen retrieval in EDTA buffer (pH 8.0) for 32 min at 98°C was performed. The blocking step was followed by incubation with Protein block buffer (Dako) for 8 min. Staining was performed in consecutive round, each round consisting of antigen retrieval, blocking, primary antibody, secondary HRP-labeled antibody, tyramide signal amplification (TSA) reagents, and antibodies denaturation. Then the primary antibodies were incubated at room temperature for 60 min, monoclonal mouse anti-human PD1 antibody (BioSB, NAT-105, 1:500), rabbit monoclonal anti-human FoxP3 (Abcam, SP97, 1:50), mouse monoclonal antibody anti-pan Cytokeratin (1:250, Clone AE1/AE3, Dako), and rabbit monoclonal antibody anti-CD8 (1:100, Clone SP16, ThermoFisher Scientific). The signal was revealed with DISCOVERY OmniMap anti-rabbit HRP (Ventana, #760-4311) or anti-mouse HRP (Ventana, #760-4310) that was incubated for 12 min. Next, TSA amplification quality controlled reagents were added: TSA Cyanine 3 (NEL744B001KT, PerkinElmer), Discovery Cy5 kit (Ventana, #760-238), Discovery RED610 kit (Ventana, #760-245), and Discovery FAM kit (Ventana, #760-243). The nuclei were visualized with Spectral DAPI (Perkin elmer, FP1490, 1:10). The images were acquired using the Vectra 3.0 (Perkin Elmer) automated quantitative pathology imaging system at 20× magnification.

### Mouse Model With TLS

*Kras^Lox-STOP-LOX-G12D/WT^* and *p53^FL ox/FLox^* mice in a C57BL6/J background purchased from the Jackson Laboratory, bred to obtain *Kras*^*Lox-STOP-LOX-G12D/WT*^*; p53^FLox/FLox^* (KP) mice and kindly provided by E. Meylan’s laboratory (EPFL-Lausanne). All mouse experiments were performed in accordance with Swiss regulations under the license numbers VD2920 and VD3242.

### Radiotherapy of KP Mice

KP tumors were initiated upon infection of lung epithelial cells with a lentiviral vector delivering Cre recombinase to activate oncogenic *Kras^G12D^* and delete *p53* (20). 13- to 16-week-old mice were infected intratracheally with 3,000 Cre-active lentiviral units. During the follow-up period, tumor volume was monitored once every 2 weeks starting from 14 weeks post lentiviral instillation. Mice were imaged using the X-rad 225CX irradiator under isoflurane anesthesia. At dedicated time points, mice underwent RT with a 20 mm^2^ collimator, optimal for whole lung irradiation at (13 mA, 3 mm Cu filter, 225 keV) 11.7 Gy with a single dose delivered in 256 s.

### Histological and Apoptosis Analysis

Tertiary lymphoid structures were quantified in non-irradiated mice at 7, 15, and 20 weeks post-instillation. Tumor apoptosis was evaluated at 20 weeks post instillation. The radiation-induced impact on the TLS was monitored 20 weeks post instillation, on mice sampled 2 h, 2 days, and 14 days post-RT. Lungs were gently insufflated with 0.5 mL of 50% PBS-50% OCT (Cell-Path ref 81-0771-00) *via* the trachea using an I.V. surflo catheter (ref SR + OX2225C1). Lobes were subsequently separated and OCT-frozen on dry ice. 20 sections of 6 µm were cut at different depths and were HE stained or stained with cleaved caspase 3 antibody for apoptosis detection (asp175, cell signaling). Images were obtained with upright microscope Axio Imager Z1 motorized and analyzed with Zen Zeiss blue software.

### Flow Cytometry and Histological Control

Mice were sampled 36 h, 14 days, and 56 days post-RT and tumors were collected and analyzed by flow cytometry as described previously (21) including gating strategy and list of antibodies. A LSRII SORP flow cytometer was used for acquisition. The quality of the tumor dissection was assessed by histology as described in the previous paragraph.

### Statistical Analysis

All results are represented as mean ± SEM. Analysis was performed using Mann–Whitney test using Prism 6 software. For Flow cytometry analysis, a minimum of 12–25 tumors per group were analyzed (coming from 4 mice minimum).

## Results

### Medullary Breast Carcinoma Display TLS

**Figure 1:**
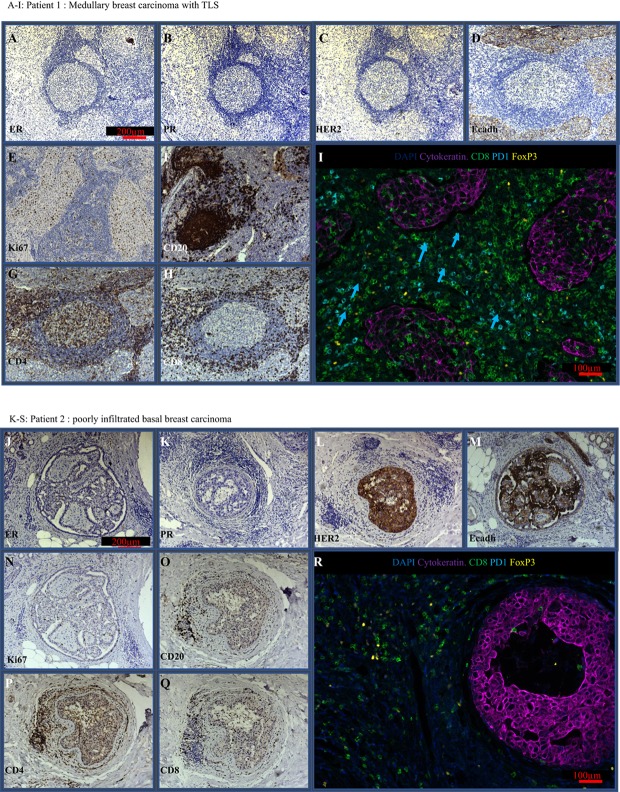

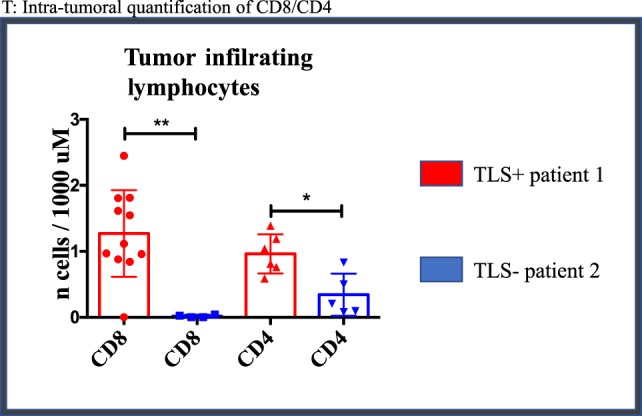
Immune infiltrate in MBC (patient 1, P1) and basal breast carcinoma (patient 2, P2). Serial histological samples of P1 and P2 were stained with routine markers. Stainings showed the following repartition: estrogen receptor [**(A–K)** ER: P1−, P2−], progesterone receptor [**(B–L)** PR: P1−, P2−], human epidermal growth factor 2 [**(C–M)** HER2: P1−, P2+], Ecadherin [**(D–N)** Ecadh: P1+, P2+], P1−, P2−], and Ki67 [**(E–O)**: P1 30% in tumor and tertiary lymphoid structures (TLS); P2 5% in tumor and 0% in immune aggregates]. Additional staining with non-routine markers was performed. They allowed characterization of TLS in P1 but not in P2: B cells [CD20 **(F–P)** P1 germinal center, P2 scarce cells], CD4 T cells [CD4 **(G–Q)** P1: present in all compartments; P2: non organized], CD8 T cells [CD8 **(H–R)** P1: restricted to the TLS periphery and invading the tumor; P2: rare in the aggregates and absent in the tumor]. IF recapitulating the key immune components, including PD1, FoxP3 CD8, and tumor cytokeratine+ are shown in **(I)** and **(S)**. PD1+ cells are absent in P2. **(T)** Intra-tumoral number of CD8 and CD4 cells in P1 and P2.

Breast cancer classification according to histo-pathological and molecular subtypes is a main diagnosis criterion and defines treatment strategy. In this study, we investigated two carcinomas from breast cancer patients: patient 1 was a triple negative subtype with basal carcinoma features (Figures 1A–D); patient 2 was HER2+, PR−, and ER− with characteristics of basal carcinoma (Figures 1K–N). Tumors from patient 1 and 2 had a proliferative index of 30 and 5%, respectively (Figures 1E,O). Upon examination, both patients displayed immune infiltration, but we noticed major differences in the organization and structures of the immune aggregates. Whereas in patient 1 >30% of the tumor area composed of well-organized TLS, patient 2 had discrete spots of immune aggregates located near the tumors in a non-organized manner.

In patient 1, the TLS were defined by the presence of a typical germinal center, showing T/B cell organization (Figures 1F–H). In addition, their proliferation index measured by Ki67 reached 30%, while aggregates in patient 2 (Figures 1P–R) had no local proliferation (Figures 1E,O). Although both tumors displayed important peritumoral CD8+ T cells (Figures 1H,R,S), only patient 1 had significant intratumoral CD8+ infiltration.

Finally, patient 1 had significant peri-tumoral infiltration in both PD1+ cells and FoxP3+ cells (Figure 1I), whereas PD1+ cells were absent in patient 2 (Figure 1S). Intra-tumoral quantification of CD8 and CD4 positive cells shows massive infiltration of CD8 and CD4 in patient 1 as compared to patient 2 (Figure 1T).

From the pathologist’s point of view, patient 1 displayed all of Ridolfi’s criteria (syncytial growth pattern in >75% of tumor area, tumor circumscription in a large capsulae, absence of intraductal component, large lymphoplasmatic infiltration, high mitotic rate, and poorly differentiated nuclear grade) and was classified as typical MBC.

Hereafter, we characterize the contribution of the TLS to the immune infiltrate and their response to radiation.

### In KP Mice, TLS Occur After Tumor Establishment and Display Common Features With TLS Observed in MBC

**Figure 2:**
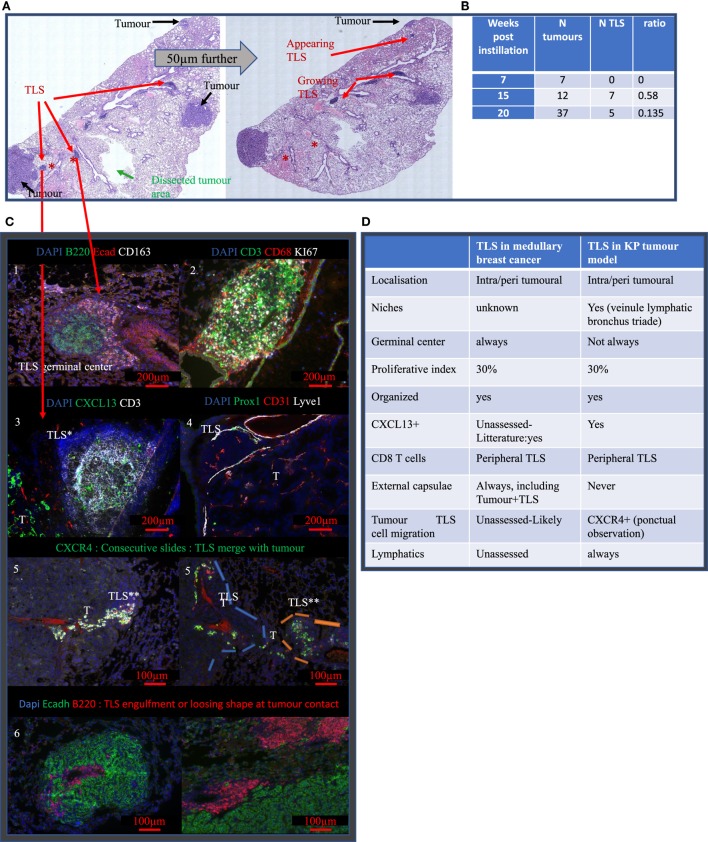
Tertiary lymphoid structures (TLS) appear after tumor onset are maintained during disease progression and share common features with TLS in MBC. **(A)** Full KP lung section H&E stained. TLS are shown by red arrows, they are located around bronchus. Analysis on serial sections is required for proper TLS detection that can be present in one section and absent in 50 µm further. Green arrow showed the area of macro-dissected tumor used for flow cytometry. **(B)** KP lungs were sampled at 7, (timing of tumor occurrence), 15, and 20 weeks (final stage) post-cre installation. Tumor and TLS quantification were performed based on H&E staining similar to **(A)**. TLS appeared after tumors’ onset. **(C)** Representative images are shown and illustrate common properties of TLS from KP and MBC such as the presence of germinal center and organizational features (1), 30% of proliferation (2). Additional stainings were performed. They show expression of CXCL13 chemokine (3), down left corner positive signal comes from the tumor (T), presence of Prox1^+^ Lyve1^+^ CD31^+^ lymphatic vessels (4). Picture 5 shows presence of aligned CXCR4^+^ cells in between TLS and tumor T. Picture 6 shows TLS engulfed by the tumor. Common features of KP versus MBC TLS are summarized in table **(D)**.

**Figure 3:**
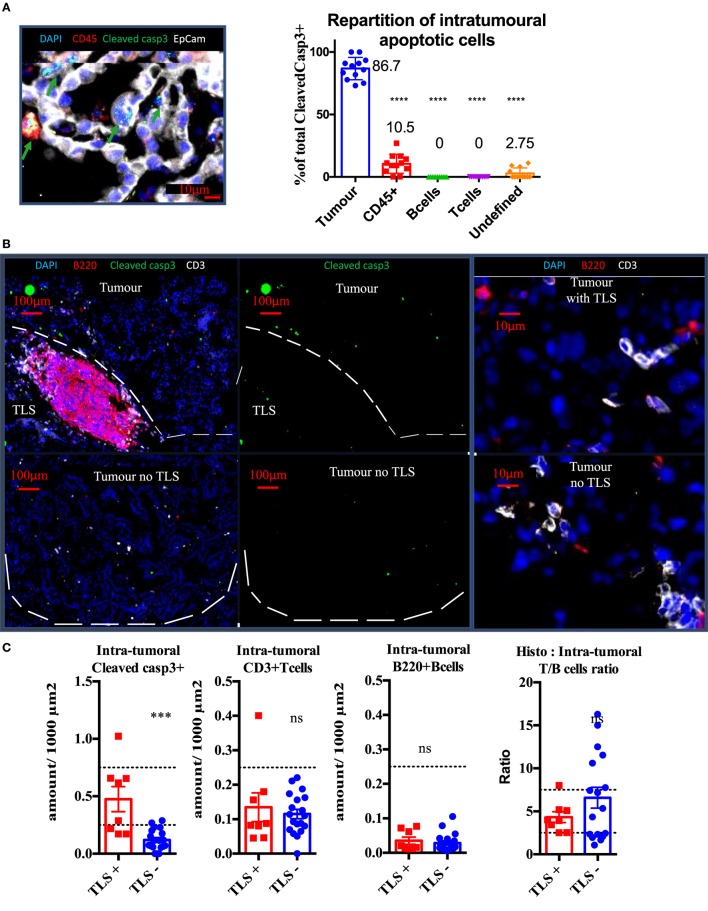
Presence of tertiary lymphoid structures (TLS) near tumors is correlated with tumor apoptosis, independently from quantitative B or T cell infiltration. Cleaved Caspase3 co-stainings with CD45 (immune), EpCap (tumor), CD3 (T cells) and B220 (B cells) apoptotic cells are mostly tumour cells **(A)**. Control tumors with or without TLS were stained to assess wether the immune contexture correlates with apoptosis (White bands delineate tumors) **(B)**. Quantification per unit surface and T/B cell ratio is shown is **(C)**.

**Figure 4:**
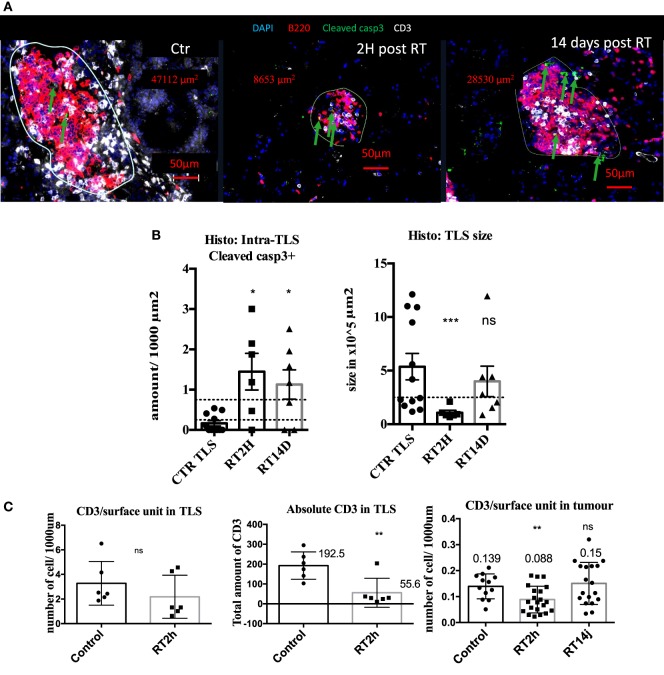
Radiation therapy (RT) increases tertiary lymphoid structures (TLS) apoptosis and their transient depletion. Apoptosis quantification measured by cleaved caspase 3 in TLS in control of KP TLS, 2 h and 14 days post-RT. TLS size was measured with Zen Zeiss software **(A)**. Quantification per surface unit is showed in **(B)**. The prevalence of CD3+ T cells was assessed in TLS and tumours to evaluate intrinsic response to RT of those cells in different compartments **(C)**.

**Figure 5:**
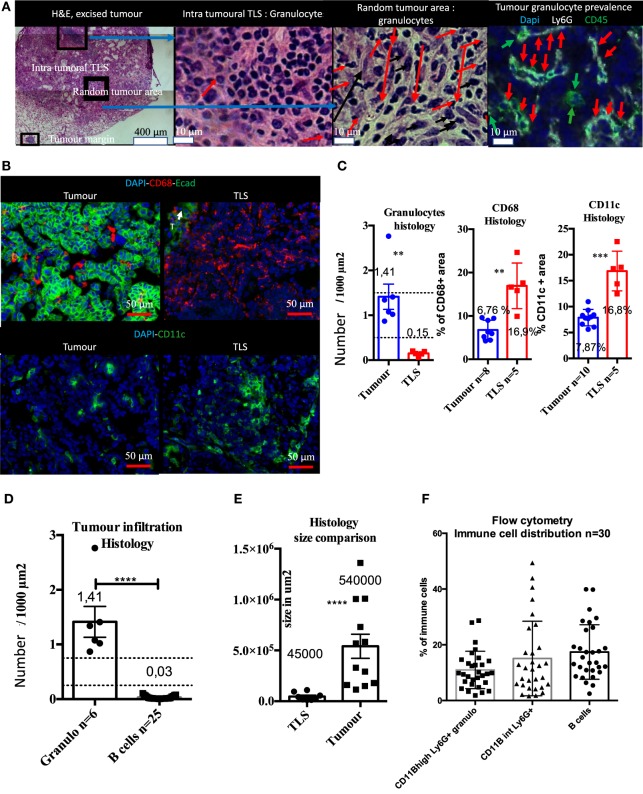
Histological and flow cytometry analysis correlation highlights granulocytes as biomarker of intra-tumor immune compartment and B cells as biomarker of tertiary lymphoid structures (TLS). Macro-dissected tumor H&E shows presence of TLS in samples. Granulocytes are almost absent in TLS and very abundant in tumors **(A)**. CD68 and CD11c+ cells that include macrophages, monocytes, and DCs, but not granulocytes, are present in both compartments but display different morphological features **(B)**. Tumor versus TLS quantification for CD68, CD11c, or granulocytes is shown in **(C)**. Granulocytes are histologically much more prevalent than B cells in the tumor compartment **(D)**. Size histological comparison of TLS and tumors is shown in **(E)**. Strong prevalence of B cells obtained by Flow Cytometry analysis suggests TLS are also analysed with this method **(F)**.

**Figure 6:**
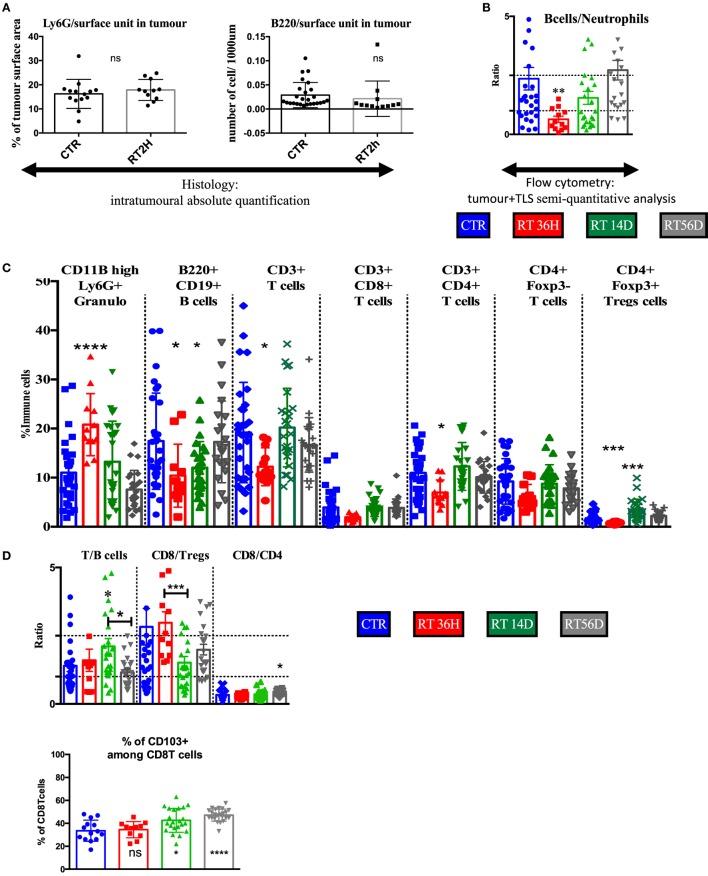
Flow cytometry analysis confirms transient depletion of tertiary lymphoid structures (TLS) following RT. **(A)** The B cells/granulocyte ratio confirms with histological analysis of the TLS depletion **(B)**. Tumor+ TLS lymphoid and granulocytes variations are presented in % of whole immune cells analyzed after macrodissection **(C)**. T/B cells, CD8/Tregs, and CD4/CD8 ratio **(D)**. Numeration of CD103 positive cells amongst CD8 cells **(E)**.

To investigate the contribution of TLS in the tumor micro-environment and their response to radiotherapy, we used KP mice in which tumors are associated with TLS. First, the number of tumors and the TLS per section was quantified at different time points (Figure 2). In KP mice, the number of tumors is determined by initial lentivirus titration, therefore, the number of tumors inside a mouse is stable over time while differentiation grade and size tend to worsen as the tumors grow. We found 2.66 tumors per mouse at W7 post-cre instillation, 4 at W15, and 9.3 at W20. This increase over time mostly reflects the increased probability to cut through tumor tissue as they increase in size. We computed the corresponding TLS/tumor ratio over the time course of the experiment and found 0 TLS at W7; 0.58 TLS/tumor at W15, and 0.135 TLS/tumors at W20. Interestingly, in bigger tumors, we observed a tendency to lose TLS shape suggesting a mechanical or spatial constraint triggered by the tumor over the TLS. The TLS in the KP model never display external capsulae, as opposed to those observed in general in MBC patient. They were eventually contoured by a crown of CD163+ Ecadh+ cells (Figure 2C, 1). The TLS proliferation rate was assessed by Ki67 staining (Figure 2C, 2) and shows a proliferation index comparable to what was seen in patient 1. They did not systematically display germinal centers. B cells were always the most prevalent cell subtype and CXCL13 was detected in TLS (Figure 2C, 3); Lymphatic vessels, determined by the triple positive staining of CD31, Prox1, and Lyve1, were systematically associated with the TLS (Figure 2C, 4). We observed possible molecular crosstalk between TLS and tumors correlated with CXCR4 and SDF-1 expression (Figure 2C, 5) and we observed pictures of TLS surrounded by tumors (Figure 2C, 6).

### Tumors With TLS Are Associated With Increased Tumor Apoptosis

Cleaved caspase 3 staining was performed on well established KP tumors in combination with EpCam (tumor cells) and CD45 (Pan-immune cells) or CD3 (T cells) and B220 (B cells). Independently from the presence of TLS, we found 86.7% of cleaved caspase 3+ signal was associated with tumor cells, 10.5% with immune cells (*p* < 0,0001), and 2.75% with unidentified stromal cells (*p* < 0.0001). Based on analysis of 15 tumors, we never found apoptotic lymphocytes in the tumor compartment. KP mice were divided into two groups, depending on the presence or absence of spatially related TLS (Figure 3B) and analyzed for tumor apoptosis using cleaved caspase3 immunostaining. The tumors associated with TLS presence showed four times fold increase in intra-tumor apoptosis (*p* < 0.001, Figure 3C) than the tumors without TLS. However, this increase was not correlated with increased CD3 T cell (Figure 3C) infiltration or with increased B220 B cells (Figure 3C). These results suggest that the TLS presence is associated with increased intra-tumor cell death and this seems independent from quantitative T/B cells tumor infiltration.

### TLS Size Is Reduced by RT

We next thought to modulate TLS presence and function with irradiation. 2 hours post radiation, we measured 8.7 times more cleaved caspase 3 cells in irradiated TLS per surface unit (*p* < 0.05 Figures 4A,B). This increase was still significant 14 days post-RT. This increase in apoptosis was associated with a transient five times size reduction of TLS (*p* < 0.001, Figure 4C), normalized 14 days post-RT. B cells absolute quantification within TLS was not monitorable because their concentration was too important. However, we found 72% significant reduction of total CD3 in TLS (Figure 4C). This reduction was no longer significant when CD3 amount was normalized by TLS’ size, suggesting the morphology of TLS directly correlates with the inner amount of T cells (Figure 4C). In tumors, we observed a 30% reduction (*p* < 0.01) of the CD3 T cells prevalence, suggesting the impact of RT on T cells is more important within TLS (Figure 4C). However, because there is no possibility to extract T cells separately from tumors and TLS, we could not perform a standard radiosensitivity assay.

### Macro-Dissected Tumors Analyzed by Flow Cytometry Contains TLS

Before analyzing the intra-tumoral immune compartment of the KP tumors by flow cytometry, we performed histological analyses on matched macro-dissected tumors. Our results showed that excised tumors contained TLS un-dissociable from the tumor core, meaning that the immune composition monitored by flow cytometry reflected both intra-tumor and the TLS components (Figure 5).

Immunofluorescence showed that Ly6G+ granulocytes were the main intra-tumoral CD45+ type of cell, whereas only few granulocytes were found in TLS (Figure 5A). CD68+ and CD11c+ myeloid cells (i.e., macrophages, dendritic cells, and monocytes) were found both in tumors and the TLS (Figure 5B). Although these markers are not sufficient to differentiate macrophages from DCs, CD68+, and CD11c+ cells had different morphology in tissue sections. They were large, round, and isolated in tumors while TLS images suggesting reticular organization (Figure 5B) that may correspond to dendritic cells. The number of granulocytes per surface unit in tumors was 10 times higher than in TLS (Figure 5C). On the contrary, surface density of CD68 and CD11c+ cells was 2.5 higher in TLS than in tumor (Figure 5C). Next, we found that histological number per surface unit of intra-tumor granulocytes was 45 times higher than B cells; at the same time, the surface of TLS was in average 12 times smaller than the surface of tumors (Figure 5E). When histological data were compared with flow cytometry, we could not confirm this difference and found a balanced number of B cells, CD11B high and intermediary granulocytes (Figure 5F). This discrepancy, taking into account the tumor/TLS size difference, suggests TLS contribution to the immune compartment analyzed by flow cytometry is major. As granulocytes were mainly found in tumors and B cells mainly in TLS, we used granulocytes as a tumor biomarker and B cells as a TLS biomarker, and postulated that the flow cytometry analysis of macro-dissected tumors was including TLS.

### TLS-Related Immune Modification After RT

As transient TLS reduction in size was shown after irradiation (Figure 4), we used it to validate the TLS contribution in the flow cytometry data of macro-dissected tumors. We used granulocytes as surrogate/biomarker of total immune compartment in tumors and B cells as surrogate/biomarker of TLS, we first quantified histologically their variation induced by RT 2 h treatment. We found no changes at these endpoints in the tumor restricted area (Figure 6A), suggesting B cells as opposed to T cells (Figure 4), tolerated radiation in the tumor bed. Opposingly, flow cytometry analysis showed a diminished B cells/granulocytes ratio by 3.7-fold with a return to basal level 14 and 56 days post-RT (Figure 6B). Correlated with results from Figure 4, this suggests TLS importantly contribute to the immune signature obtained from excised tumors. Granulocyte proportion was enhanced at 36 h and then returned to basal level (Figure 6C). 36 h post-RT, B cell proportion was reduced by 35% (*p* < 0.05, Figure 6C), T cells by 30% (*p* < 0.05, Figure 6C), and CD4T cells by 37% (*p* < 0.05, Figure 6B). Total T cells were reduced: among CD4+ T cells, FoxP3 expressing Treg displayed a 60% reduction (*p* < 0,001, Figure 6C), while CD8 and CD4 non-Treg reduction was non-significant (Figure 6C), although a trend toward a decrease was observed. 14 and 56 days post-RT, to the exception of Tregs that showed a transient increase (+200%, *p* = 0.0001, Figure 6C), all variations in cell proportion returned to normal consistently with histological normalization of the TLS morphology. In contrast to the B cell/granulocyte ratio, the inter-lymphoid ratio remained stable at early endpoint post-RT (Figure 6D) suggesting subpopulation among lymphoid cells responded in a common way to radiation. However, during the restoration phase of the TLS, CD8 repopulation was significantly counterbalanced by Tregs enrichment 14 days post-RT (Figure 6D) but associated with enrichment of CD103 positive cells among CD8 cells at late time point (Figure 6E).

## Discussion

We used two case reports to highlight a major difference in immune infiltration within two basal breast carcinomas in order to illustrate the need for characterization of peri-tumoral immune contexture as these markers are not routinely performed in the clinic. While in MBC patient 1, the TLS are composed of locally proliferating lymphocytes organized around germinal centers, in patient 2 common aspecific aggregates are composed of non-proliferative recruited lymphocytes. These two sources of lymphocytes might not infiltrate tumors nor respond to loco-regional treatment the same way. This question is highly relevant for radio-oncologists as the TLS might contain potent lymphoid precursors that could be modulated (activated or depleted) by irradiation. Then, we used an experimental model with functional TLS (KP mice) to investigate the response of TLS to RT. Correlating histological analysis and previously established (21) flow cytometry method, we developed a semi-quantitative and qualitative analysis of TLS composition and assessed their response to RT. Main findings showed (1) increased apoptosis in tumors associated with TLS; (2) confirmed B cells as the main biomarker of TLS in patients and KP model; (3) highlighted TLS sensitivity to RT *in vivo*; and (4) highlighted plasticity of TLS and their reappearance 14 days post-RT, providing a window of opportunity to optimize their function. Our results are a first step toward a comprehensive characterization of the composition of TLS after RT. In the era of immunotherapy combined with RT, TLS might be the next attractive target.

Investigating tumor physiology through its micro-environmental biology is an attractive angle for treatment optimization. Understanding the micro-environmental imprint and its potential stereotypical response to classical anti-cancer strategies may help to define broader therapeutic combination options which could be less resistant (22) and consistent with the increasingly common paradigm of “personalizing medicine.” Indeed, some micro-environmental characteristics are rare within a subgroup of defined specific tumor patients but may be studied and better understood if present in other more frequent primary tumor subtypes. In this context, TLS have shown a common physiological pattern, which is independent from the primary tumor subtype/organs and may stimulate common strategies applicable to various cancers (10, 23). Our study highlights the relevance of TLS and show several similarities between the TLS located in MBC patients and in the experimental KP mice. They both include about 30% lymphoid proliferative rate; presence of germinal centers with B cells as a major cell subtype; evidence of organized architecture; and presence of Tregs. Whereas B cells have an initiating role for TLS formation in autoimmune, transplantation-related and infectious diseases (24, 25) their role in the field of cancer remains to be clarified. Interestingly, the major difference between the TLS in MBC and KPs was the absence of external capsulae surrounding the tumors and the TLS in the mouse model. Lymph node metastasis occurs in 50% of the cases in KP mice at late stage, whereas MBC patients are less prone to metastasis compared to other breast basal carcinoma patients. In MBC, the presence of the external capsulae might protect from local and distant dissemination. In the KP mice, the presence of the TLS was associated with enhanced intra-tumor apoptosis without enhanced T/B cell infiltration, suggesting a qualitative regulation of intra-tumor lymphocytes related to TLS.

Current knowledge about TLS behavior in the context of treatment is inexistent. We hypothesize that MBC would be an ideal type of tumor to clinically investigate this question as they present strong lymphoplasmocytic infiltration, commonly organized in TLS; however, the presence of the TLS is not a deciphering criterion for any tumor type. The purpose of our study was to stimulate this research field by providing a first highlight of the interaction between TLS and tumors in the context of RT. After RT, the TLS showed an acute increase of apoptosis and size reduction. While size tends to normalize after 2 weeks, the apoptotic rate remained high, suggesting active and continuous proliferation within residual irradiated cells. In order to decipher the impact of RT on TLS composition, we used flow cytometry-based methodology and combined it with sequential examination of the composition of the TLS over the course of RT. As B cells are mainly found in TLS and granulocytes mainly in tumors, we used the B cell/granulocyte ratio as a marker of TLS/tumor abundance. We found indeed a significant decrease in the B cell/granulocyte ratio at acute time point post-RT consistent with histological finding and confirming TLS contribution to immune profile of macro-dissected tumors. We reported sequential enrichment first in CD8 followed by Foxp3 Tregs in macro-dissected tumors post-RT. Interestingly, Foxp3 Tregs enrichment corresponds to the restoration phase of the TLS. Consistently, immunosuppressive function of specific Tregs located in the TLS has been suggested in KP mice (9) and in breast cancer patients with TLS in whom FoxP3/T cells were shown to be the only marker associated with poor prognosis (12). On the other hand, at late time point a highly significant increase in CD103 positive cells among CD8 was measured, suggesting infiltration with memory T cells (26).

The KP model was first published in 2009 (20) and considered as “non-immunogenic” due to its low level of basal infiltrating immune cells. It was subsequently modified to express OVA peptide and stimulate immune response upon adoptive T cell transplantation, but this strategy only led to minor improvement (27). However, a better outcome was obtained with combination of chemotherapy and immune checkpoint inhibitors (15) and interestingly, this strategy was found to be TLS dependent. More recently, Faget et al. described occurrence of TLS in the native KP model and achieved an immune-based tumor delay mediated by normalization of tumor vasculature, using a granulocytes depleting antibody in combination with immune checkpoint blockade (21). In the meantime, a vascular-based strategy also showed TLS occurrence in a pancreatic cancer model, associated with tumor delay (14). This suggests TLS formation or functionality may be dependent on vascular quality and may be of importance to design combined therapeutic strategy.

In conclusion, we investigated the radiation-induced modification of TLS composition and tumor immune infiltrate over time. Although descriptive, our study provides the first timely comprehensive view of TLS modulation occurring in tumors associated with TLS after irradiation. In the context of tumors associated with TLS, flow cytometry analysis includes immune infiltrate coming from both tumors and TLS. The radiation-induced response is complex. It induces a reset of the TLS followed by a rapid restoration associated with Tregs enrichment of the global tumor microenvironment. Future experiments are needed to enhance the radiation-induced beneficial components as well as repress the deleterious ones. More specifically, comparing TLS versus lymph node response to radiotherapy would increase our understanding of the loco-regional immune response. In addition, neoadjuvant irradiation of the breast at lower doses in the context of TLS may have immunomodulatory properties and enhance systemic immunity. Such strategies should bring insights for personalized therapeutic management of tumors associated with TLS. In the specific context of MBC where TLS are commonly observed, acute manipulation of TLS composition using RT combined with immune checkpoint inhibitor prior mastectomy could provide long-lasting benefits and be worth further clinical investigation.

## Ethics Statement

Experiments were performed in accordance with Swiss regulations under the license numbers VD2920 and VD3242.

## Author Contributions

GB, GL, JB, and M-CV designed the project, analyzed data, and wrote the manuscript. GB, PK, JF, EM, SR, and PM-G organized, performed, and analyzed most experiments including animal sampling and the daily follow-up of animals. GL perfomed histological analysis of human samples. All authors approved the final version of the manuscript.

## Conflict of Interest Statement

GL was employed by the company Unilabs. All other authors declare no conflict of interest. The reviewer MG and handling Editor declared their shared affiliation.
